# Impact of coronary CT angiography in selection of treatment modalities and subsequent cardiovascular events in Thai patients with stable CAD

**DOI:** 10.1007/s00392-023-02313-1

**Published:** 2023-10-04

**Authors:** Thosaphol Limpijankit, Sutipong Jongjirasiri, Krissada Meemook, Nattawut Unwanatham, Sasivimol Rattanasiri, Ammarin Thakkinstian, Jiraporn Laothamatas

**Affiliations:** 1 Division of Cardiology, Department of Medicine, Faculty of Medicine, Ramathibodi Hospital, Mahidol University, 270 Rama VI Road, Ratchathewi, Bangkok, 10400 Thailand; 2 Department of Diagnostic and Therapeutic Radiology, Faculty of Medicine, Ramathibodi Hospital, Mahidol University, Bangkok, Thailand; 3 Department of Clinical Epidemiology and Biostatistics, Faculty of Medicine, Ramathibodi Hospital, Mahidol University, Bangkok, Thailand; 4 Faculty of Heath Science Technology, Chulabhorn Royal Academy, Bangkok, Thailand

**Keywords:** Coronary computed tomography angiography, Aspirin, Statin, Revascularization, CV outcomes

## Abstract

**Background:**

Coronary computed tomography angiography (CCTA) enables improved diagnosis of subclinical, coronary artery disease (CAD). This retrospective cohort study investigated the association between different treatment modalities guided by CCTA and the prevention of major adverse cardiovascular events (MACEs) in patients with stable CAD.

**Methods:**

From 2005 to 2013, a total of 9338 patients, including both asymptomatic individuals with risk factors and symptomatic patients with suspected CAD, who underwent CCTA were analyzed. The patients were categorized into one of three groups based on results of CCTA: obstructive CAD (≥ 50% stenosis in at least one vessel), non-obstructive CAD (1–49% stenosis in at least one vessel), and no observed CAD (0% stenosis). They were subsequently followed up to assess the treatment they received and the occurrence of MACEs (cardiovascular death, non-fatal myocardial infarction, non-fatal stroke, or late revascularization).

**Results:**

During an average follow-up period of 9.9 ± 2.4 years, patients with obstructive CAD had the highest incidence of MACEs (19.8%), followed by those with non-obstructive CAD and no coronary artery stenosis (10.3 and 5.5%, respectively). After adjusting for confounding variables, it was found that patients treated with statins alone were the least likely to develop MACEs in all three groups, compared to those receiving no treatment, with hazard ratios (95% CI) of 0.43 (0.32, 0.58), 0.47 (0.34, 0.64), and 0.46 (0.31, 0.69), respectively. In patients with obstructive CAD, treatment with a combination of statin and aspirin, or early revascularization was associated with a lower likelihood of experiencing MACEs compared to no treatment with hazard ratios of 0.43 (0.33, 0.58) and 0.64 (0.43, 0.97), respectively.

**Conclusion:**

CCTA offers useful guidance for the treatment of patients with stable CAD and shows potential for prevention of CV events. However, the full validation of a given strategy utilizing CCTA will require a prospective longitudinal study, utilizing a randomized clinical trial design.

**Supplementary Information:**

The online version contains supplementary material available at 10.1007/s00392-023-02313-1.

## Introduction

Coronary artery disease (CAD) remains the leading cause of cardiac sudden death worldwide [1]. The detection of subclinical atherosclerotic CAD is now possible, allowing for the identification of high-risk patients and early initiation of primary prevention [2, 3]. Coronary computed tomography angiography (CCTA) is the only non-invasive test that can visualize coronary pathology, including coronary artery calcium, stenotic lesions, and their extent. Recent studies have shown that CCTA is effective as a first-line diagnostic test for screening patients suspected of having symptomatic CAD, guiding treatment, and thereby reducing the risk of future myocardial infarction (MI) and stroke [4, 5].

The Scottish Computed Tomography of the Heart (SCOT-HEART) trial [6] demonstrated that adding CCTA to the standard-of-care for low-to-intermediate risk patients with chest pain can enhance clinical decision-making, reduce the need for invasive coronary angiography (CAG) and improve clinical management, leading to better patient outcomes. Based on a 5-year follow-up study, they reported a 41% lower risk of nonfatal MI or cardiovascular (CV) death when CCTA was added to standard care alone [7]. Moreover, findings from the Prospective Multicenter Imaging Study for Evaluation of Chest Pain (PROMISE) trial [8] have substantiated the efficacy of CCTA in detecting non-obstructive CAD. This discriminative ability of CCTA for event prediction surpasses that of functional testing. Overall, CCTA exhibits better risk stratification capability, resulting in improvements in accuracy, safety, and the utilization of preventive medications. As evidence continues to accumulate, cardiovascular associations have released preliminary guidelines for first-line use of CCTA for the diagnosis of CAD in symptomatic patients [9–11].

In contrast, current guidelines for management of asymptomatic patients generally do not recommend using CCTA to screen for CAD due to the potential for use of unnecessary drugs and further procedures, including revascularization, without proven benefit [12, 13]. Despite this, many institutions routinely perform CCTA examinations of asymptomatic individuals with risk factors as part of screening programs. This poses a challenge in determining appropriate management based on the CCTA findings.

Given the current literature, it remains unclear how to best utilize CCTA findings to guide treatment in both asymptomatic individuals with risk factors and symptomatic patients with suspected stable CAD. Current clinical practice guidelines primarily consider the coronary artery calcium score (CACS) as a useful test for personalizing statin therapy allocation [14, 15] and initiating (or deferring) antiplatelet therapy for primary prevention [16, 17]. The extent to which CCTA findings can enhance physician judgement and influence long-term outcomes remain uncertain. Therefore, this study was conducted to investigate the association between different treatment modalities guided by CCTA findings and the prevention of long-term major adverse CV events (MACEs) in both asymptomatic individuals with risk factors and symptomatic patients with stable CAD.

## Materials and methods

This retrospective cohort study focused on consecutive patients who underwent CCTA for CAD assessment at the Advanced Diagnostic Imaging Center (AIMC), Ramathibodi Hospital, Mahidol University, between November 2005 and November 2013. The study protocol was approved by the Ethics Committee of the Faculty of Medicine, Ramathibodi Hospital, Mahidol University (# COA.MURA2019/758). Written informed consent was obtained from each participant before performance of the CCTA.

The criteria for inclusion in the study were: (1) age > 18 years; (2) a moderate to high atherosclerotic cardiovascular disease (ASCVD) risk score [18] or mild chest symptoms suspected to be caused by stable CAD. Exclusion criteria were: (1) history of prior coronary artery bypass surgery (CABG) or percutaneous coronary intervention (PCI); (2) CV events (such as MI or stroke) or revascularization; (3) high serum creatinine (> 1.5 mg/dL); (4) history of severe seafood or contrast allergy.

The history and physical examination of each patient done prior to CCTA provided information on demographics (age, sex), risk factors (smoking, diabetes mellitus [DM], hypertension and hypercholesterolemia), body mass index (BMI, kg/m^2^), waist circumference, and medications (prior and current). Abnormal waist circumference was defined as ≥ 90 cm (36 inches) in men and ≥ 80 cm (32 inches) in women. Laboratory data included fasting plasma glucose (FPG), lipid profile (triglyceride, low-density lipoprotein cholesterol [LDL-C], high-density lipoprotein cholesterol [HDL-C]), serum creatinine, and uric acid. DM was defined as overnight FPG ≥ 126 mg/dL or taking anti-diabetic medication. Hypertension was defined as systolic BP (SBP) ≥ 140 mm Hg and/or diastolic BP (DBP) ≥ 90 mm Hg or taking anti-hypertensive medication. Hypercholesterolemia was defined as total cholesterol ≥ 200 mg/dL or LDL-C ≥ 130 mg/dL or taking a statin medication. Smoking status was classified as current smoking, ex-smoking (stopped for more than 1 month), or never smoked. The estimated glomerular filtration rate (eGFR, mL/min/1.73m^2^) was calculated using the Chronic Kidney Disease Epidemiology Collaboration (CKD-EPI) equations. Chronic kidney disease (CKD) was defined as an eGFR < 60 mL/min/1.73 m^2^.

As part of the protocol, all patients also underwent measurement of arterial stiffness using the cardio-ankle vascular index (CAVI) on the same day as the CCTA study. This measurement was performed using a Vasera VS-1000 vascular screening system (Fukuda Denshi, Japan) and detailed previously [19, 21]. The mean values of the right and left CAVIs were used for analysis. According to the manufacturer, values < 8 are considered as normal, 8 to < 9 are borderline, and ≥ 9 are high, suggesting the presence of arteriosclerosis [20, 21]. A high CAVI value (≥ 9) was considered as a candidate risk factor for atherosclerosis in patients with suspected CAD.

### Coronary CT angiographic scanning

Multidetector CT angiographic scans were performed using a 64-slice CT scanner (Somatom Sensation 64 eco, Siemens, Forchheim, Germany) before 2008 and a 320-slice CT scanner (Aquilion ONE, Toshiba, Tokyo, Japan) after 2008. Two coronary CT scan findings were used in the analysis: (1) CACS; (2) degree and location of stenoses. The CACS was calculated using the Agatston method with a commercially available external workstation (Vitrea fx 3.0.1, Vital Images, Minnesota, USA). It was obtained by summing the individual lesion scores in all coronary arteries and categorized into four groups: 0, 1–99, 100–399 and ≥ 400, interpreted as no identifiable plaque, mild, moderate, or extensive atherosclerotic plaque, respectively.

The degree of coronary stenoses was evaluated after injecting 70–90 mL of radiocontrast material (Ultravist 370 mgI/mL, Bayer Healthcare, NJ, USA) through the right basilic vein using an 18-gauge intravenous catheter, followed by a 20 mL saline flush at a flow rate of 5 mL/sec. Automated bolus tracking was used to synchronize the arrival of the contrast media and the scan. Images were acquired during an inspiratory breath hold of 5–10 s, starting four seconds after the injection.

Three-dimensional reconstructions and cross-sectional imaging measurements were performed for four epicardial coronary arteries (left anterior descending, left circumflex, right coronary and left main arteries). Coronary stenoses were classified into three groups: obstructive CAD (≥ 50% stenosis in at least one vessel), non-obstructive CAD (1–49% stenosis in at least one vessel), and no coronary artery stenosis (0% stenosis in all vessels).

### Data collection

The data from case record forms (i.e., demographic information, blood tests, CAVI, and the full report of the coronary CT scan) were entered into electronic databases twice, by two independent healthcare personnel who were trained catheterization laboratory nurses and AIMC staff. The entered data were meticulously cross-checked and adjusted to ensure consistency and accuracy. Subsequently, the electronic databases were exported to Excel spreadsheets for further statistical analyses.

### Treatment and clinical follow-up after coronary CTA

Following the CCTA study, patients received treatment based on their own physician’s discretion, utilizing the official report of CCTA, arterial stiffness measured by CAVI, and baseline patient characteristics. Treatment options comprised statins and/or aspirin therapy in combination with lifestyle modification and risk factor management. Additionally, some patients underwent cardiac stress testing, invasive CAG, and/or early revascularization. For this analysis, follow-up data on treatment, clinical outcomes, and vital status were retrieved until the year 2019. Cross-sectional data at baseline CTA measurement were linked with four ongoing data sources: (1) electronic medical records of the Division of Information Technology, Ramathibodi Hospital, (2) 43-file data from the Strategy and Planning Division, Office of the Permanent Secretary, Ministry of Public Health (MoPH), (3) Information and Communication Technology Center, MoPH, and (4) Central Office for Healthcare Information. Codes from the International Classification of Diseases (ICD)-10 and ICD-9 were used to identify specific outcomes of interest, such as fatal and non-fatal MIs (non-ST-segment or ST-segment elevation MIs), fatal and non-fatal strokes, and late or repeated revascularizations. The primary outcome of interest was the occurrence of MACEs, which encompassed CV death, non-fatal MI, non-fatal stroke, and late revascularization. In case a patient experienced multiple CV events, only the first event was used for analysis. Secondary outcomes included individual MACE outcomes and bleeding complications, such as gastrointestinal (GI) and intracranial bleeding.

### Statistical analysis

Baseline characteristics were summarized as mean ± SD for continuous variables and as percentages for categorical variables. A two-sided Student’s *t* test, Chi-square test, or quartile regression, as appropriate, was used to compare these characteristics among different degrees of stenosis and treatment modalities in relation MACE outcomes. The cumulative incidence of MACEs was calculated for each treatment group, considering all causes of death as competing risk events using the Fine and Gray method and stratified by CAD groups [22]. To assess the effects of treatment modalities on subsequent MACEs, a multivariate cause-specific Cox hazard (CSH) regression was performed with the following steps. First, univariate analyses were performed, considering potentially confounding variables (age, sex, BMI, abnormal waist circumference, smoking status, HT, DM, CKD, LDL-C, HDL-C, triglyceride, uric acid, ABI, CAVI, CACS, and the number of stenotic vessels) in the CSH model. Secondly, significant confounding variables were simultaneously included in the final model. Hazard ratios (HRs) and their corresponding 95% confidence intervals (CIs) were estimated and reported for each confounding variable. Furthermore, cumulative incidence curves for MACEs were constructed and compared among different treatment modalities. All analyses were performed stratifying by obstructive CAD (i.e., ≥ 50%, 1–49%, and 0% stenosis) using STATA 17.0. (Stata Statistical Software: Release 17; StataCorp, TX, USA). A *p* value of less than 0.05 was considered statistically significant.

## Results

A total of 9338 patients were included in this retrospective analysis, categorized into three groups based on the degree of coronary stenosis determined by CCTA. The numbers of patients with obstructive CAD, non-obstructive CAD and no coronary artery stenosis were 1788 (19.1%), 4030 (43.2%) and 3520 (37.7%), respectively. Patients with obstructive CAD were more likely to be older than 60 years, male and overweight (BMI ≥ 23). They also tended to have traditional risk factors such as smoking (current or ex-smoker), hypertension, DM, CKD, LDL-C level ≥ 100, HDL-C level < 40, triglyceride level ≥ 150, and elevated uric acid. Additionally, they more frequently had CAVI values ≥ 9 (Table 1). The occurrence of CACS values ≥ 400 or in the range of 100–399 was also more frequent in the obstructive CAD group.

**Table 1:** Baseline characteristics and treatment modalities grouped by degree of coronary stenosis on CCTA

	Obstructive CAD (≥ 50%)	Non-obstructive CAD (1–49%)	No stenosis (0%)	*p* value
	*N* = 1788	*N* = 4030	*N* = 3520	
Age group, %				
≥ 60	65.7	49.9	34.7	< 0.001
≥ 45 to < 60	33.4	46.5	59.3	
< 45	1.0	3.6	6.0	
Male sex, %	55.0	37.7	27.3	< 0.001
BMI ≥ 23, %	74.9	69.8	65.1	< 0.001
Abnormal waist circumference, %	51.4	51.2	48.0	0.010
Current/ex-smoker, %	22.7	13.2	11.0	< 0.001
HT, %	80.6	66.5	50.9	< 0.001
DM, %	36.8	26.6	19.3	< 0.001
CKD (eGFR < 60), %	12.2	8.0	4.1	< 0.001
LDL-C ≥ 100 mg/d, %	75.3	80.3	83.7	< 0.001
HDL-C < 40 mg/dL, %	21.8	16.2	12.2	< 0.001
Triglyceride ≥ 150 mg/dL, %	30.7	27.1	23.1	< 0.001
Uric acid ≥ 7 mg/dL, %	18.4	12.9	10.8	< 0.001
ABI ≤ 0.9, %	1.9	1.9	2.6	0.150
CAVI ≥ 9, %	47.6	40.2	28.6	< 0.001
Number of stenotic vessels, %				
3 vessels/LM	13.8	0.0	0.0	< 0.001
2 vessels	22.9	0.0	0.0	
1 vessel	63.3	0.0	0.0	
CACS, %				
≥ 400	24.9	2.3	0.1	< 0.001
100–399	34.8	12.3	1.0	
1–99	30.1	48.4	16.6	
0	10.2	37.0	82.3	
CAG, %	26.1	7.3	2.4	< 0.001
Cardiac stress test, %	5.1	1.6	0.5	< 0.001
Treatment after CCTA, %				
No-medication	24.8	32.7	39.6	< 0.001
ASA	1.8	1.6	1.4	
Statin	30.8	40.9	45.3	
ASA & Statin	35.4	24.4	13.6	
Early revascularization	7.3	0.4	0.0	

*ABI* ankle-brachial index, *ASA* aspirin, *BMI* body mass index (kg/m^2^), *CACS* coronary artery calcium score, *CAG* coronary angiogram, *CAVI* cardio-ankle vascular index, *CCTA* coronary computed tomography angiography, *CKD* chronic kidney disease, *eGFR* estimated glomerular filtration rate (mL/min/1.73m^2^), *DM* diabetes mellitus, *HT* hypertension, *HDL-C* high-density lipoprotein-cholesterol, *LDL-C* low-density lipoprotein-cholesterol, *LM* left main

Most patients were initially managed with medication [*n* = 6035 (64.6%)]. Some were further evaluated with cardiac stress testing [*n* = 171 (1.8%)] or underwent CAG [*n* = 845 (9.0%)]. Subsequently, patients were classified into five treatment groups. The majority (63.0%) were treated with either statins alone (40.6%) or a combination of statin and aspirin (22.4%). Other treatment groups were characterized based on risk factor modification without medication (33.8%), early revascularization (1.6%), and aspirin alone (1.6%). Compared to the baseline medication before CCTA, there was a significant increase in the percentage of patients receiving statin (from 9.4 to 63%) and aspirin (from 0.5 to 24%) therapies.

Patients with obstructive CAD were more frequently treated with a combination of statin and aspirin compared to those with non-obstructive CAD or no coronary artery stenosis (35.4, 24.4 and 13.6%, respectively). Similarly, the utilization of early revascularization was higher in patients with obstructive CAD (7.3%) compared to those with non-obstructive CAD (0.4%) or no coronary artery stenosis (0%) (Table 1). Conversely, patients without coronary artery stenosis were more likely to be treated with either statins alone (45.3%) or lifestyle modifications and risk factor management without medication (39.6%). Notably, the use of aspirin alone was uncommon in all three groups (1.4–1.8%).

### Long-term clinical outcomes

During the follow-up period which averaged 9.9 ± 2.4 years, a total of 965 patients (10.3%) experienced MACEs, including CV death (7.2%), non-fatal MI (2.4%), non-fatal stroke (6.2%) or late revascularization (3.4%).

The incidence of MACEs varied across age groups. Patients aged over 60 years had the highest incidence of MACEs, followed by those between 45 and 60 years, and younger than 45 years, with rates of 14.7, 6.0 and 4.3%, respectively.

Patients with obstructive CAD had a higher frequency of MACEs (19.8%). This was primarily driven by late revascularization, non-fatal stroke, and non-fatal MI (10.5, 8.1 and 6.4%, respectively; see Supplemental Table 1). In contrast, MACEs were less frequent in the non-obstructive CAD and no coronary artery stenosis groups (10.3 and 5.5%, respectively).

In terms of anatomical involvement, patients with triple-vessel and left main disease had the highest incidence of MACEs, followed by 2-vessel and 1-vessel CAD, with rates of 31.4, 24.4, and 9.1%, respectively. Among these, left main disease was associated with the highest frequency of MACE (50%), while the percentages for LAD, RCA and LCX were 32.2, 26.9 and 24.9%, respectively.

The frequency of bleeding events (specifically GI or intracranial bleeding) was 2.5%, primarily associated with the use of aspirin or revascularization. Bleeding events occurred most frequently in the obstructive CAD group, followed by the non-obstructive CAD and no stenosis groups (4.0, 2.7 and 1.6%, respectively).

### Predictors of MACE occurrence

In the univariate analysis, hypertension, DM, CKD, CAVI and CACS each showed a significant association with MACEs in all three CAD stenosis groups (see Table 2). Other risk factors demonstrated inconsistent associations among these three groups; these included old age, male sex, BMI ≥ 23, abnormal waist circumference, smoking status, uric acid, LDL-C, and HDL-C. Notably, LDL-C level ≥ 100 and HDL-C level < 40 were associated with significant reductions in MACEs (a protective effect), specifically in the non-obstructive CAD group. This counter-intuitive finding may be attributed to the frequent use of statins in our cohort following baseline blood collection.

**Table 2:** Incidence and hazard ratios of MACE occurrence in follow-up by characteristics and treatment modalities grouped by degree of coronary stenosis: univariate

	Obstructive CAD (≥ 50%), *n* = 1788			Non-obstructive CAD (1–49%), *n* = 4030			No stenosis (0%), *n* = 3520		
	Incidence/1000 patient-years	HR (95% CI)	*p* value	Incidence/1000 patient-years	HR (95% CI)	*p* value	Incidence/1000 patient-years	HR (95% CI)	*p* value
Treatments									
Early-revas	35.58	1.13 (0.77, 1.67)	0.533	52.42	5.14 (2.22,1.89)	< 0.001			
ASA + Statin	19.39	0.62 (0.47, 0.81)	0.001	14.98	1.47 (1.16, 1.86)	0.001	14.92	2.97 (2.13, 4.15)	< 0.001
Statin	16.53	0.52 (0.39, 0.70)	< 0.001	7.26	0.71 (0.55, 0.91)	0.007	3.04	0.61 (0.42, 0.89)	0.011
ASA	30.81	0.98 (0.53, 1.84)	0.959	13.29	1.29 (0.65, 2.53)	0.456	18.68	3.74 (1.87, 7.49)	< 0.001
No-medication	32.27	1		10.26	1		5.05	1	
Age group									
≥ 60	26.19	4.80 (0.68, 33.80)	0.115	15.01	2.73 (1.41, 5.30)	0.003	8.94	3.78 (1.68, 8.54)	0.001
≥ 45 to < 60	17.02	3.10 (0.44, 21.98)	0.257	6.17	1.10 (0.56, 2.17)	0.776	4.21	1.70 (0.75, 3.85)	0.203
< 45	5.59	1		5.68	1		2.65	1	
Sex									
Male	26.32	1.42 (1.15, 1.76)	0.001	13.59	1.61 (1.33, 1.95)	< 0.001	6.36	1.15 (0.85, 1.56)	0.374
Female	18.40	1		8.42	1		5.40	1	
BMI									
≥ 23	24.06	1.30 (1.00, 1.67)	0.047	10.68	1.12 (0.90, 1.39)	0.299	6.26	1.38 (1.01, 1.88)	0.041
< 23	18.44	1		9.51	1		4.61	1	
Waist circumference									
Abnormal	23.84	1.12 (0.91, 1.38)	0.292	11.50	1.26 (1.04, 1.54)	0.018	5.39	0.99 (0.74, 1.32)	0.922
Normal	21.56	1		9.2	1		5.92	1	
Smoking status									
Current/ex-smoker	26.79	1.25 (0.99, 1.59)	0.063	13.51	1.35 (1.05, 1.74)	0.020	7.11	1.26 (0.84, 1.89)	0.261
Never smoked	21.40	1		9.91	1		5.51	1	
HT									
Yes	26.78	3.62 (2.39, 5.48)	< 0.001	14.32	4.98 (3.60, 6.89)	< 0.001	8.04	2.47 (1.81, 3.37)	< 0.001
No	7.37	1		2.89	1		3.29	1	
DM									
Yes	31.14	1.73 (1.41, 2.12)	< 0.001	17.69	2.27 (1.87, 2.76)	< 0.001	10.23	2.25 (1.67, 3.02)	< 0.001
No	18.00	1		7.81	1		4.61	1	
CKD									
eGFR < 60	34.48	1.62 (1.24, 2.12)	0.001	24.65	2.72 (2.10, 3.52)	< 0.001	16.58	3.30 (2.12, 5.14)	< 0.001
eGFR ≥ 60	21.18	1		9.27	1		5.23	1	
LDL-C									
≥ 100	22.93	1.00 (0.78, 1.29)	0.970	9.28	0.58 (0.46, 0.72)	< 0.001	5.49	0.83 (0.57, 1.22)	0.343
< 100	22.95	1		15.91	1		6.53	1	
HDL-C									
≥ 40	21.86	0.84 (0.66, 1.07)	0.171	9.97	0.76 (0.60, 0.97)	0.030	5.33	0.72 (0.49, 1.06)	0.093
< 40	26.52	1		13.27	1		7.84	1	
Triglyceride									
≥ 150	21.22	0.90 (0.71, 1.14)	0.377	12.62	1.29 (1.04, 1.60)	0.019	6.67	1.26 (0.91, 1.76)	0.164
< 150	23.69	1		9.76	1		5.38	1	
Uric acid									
≥ 7	30.43	1.42 (1.10, 1.83)	0.006	15.60	1.59 (1.23, 2.05)	< 0.001	7.76	1.42 (0.94, 2.16)	0.096
< 7	21.42	1		9.84	1		5.43	1	
Ankle brachial index									
ABI ≤ 0.9	27.23	1.22 (0.59, 2.51)	0.587	12.29	1.16 (0.55, 2.46)	0.698	6.82	1.27 (0.52, 3.13)	0.596
ABI > 0.9	22.50	1		10.78	1		5.81	1	
CAVI									
≥ 9	26.28	1.36 (1.09, 1.70)	0.007	16.00	2.17 (1.75, 2.68)	< 0.001	8.56	1.71 (1.28, 2.28)	< 0.001
< 9	19.17	1		7.30	1		4.59	1	
CACS									
≥ 400	35.85	4.56 (2.64, 7.89)	< 0.001	35.87	5.70 (3.59, 9.05)	< 0.001			
100–399	26.70	3.41 (1,98, 5.88)	< 0.001	14.60	2.34 (1.67, 3.28)	< 0.001	5.68	1.02 (0.25, 4.21)	0.982
1–99	14.51	1.86 (1.05, 3.28)	0.033	9.14	1.44 (1.11, 1.89)	0.007	9.07	1.77 (1.28, 2.44)	< 0.001
0	7.85	1		6.50			5.09	1	
Number of stenotic vessels									
3 vessels/LM	41.05	2.37 (1.81, 3.10)	< 0.001						
2 vessels	28.99	1.67 (1.31, 2.13)	< 0.001						
1 vessel	17.27	1							

Abbreviations as in Table 1

*HR* hazard ratios, *MACEs* major adverse cardiovascular outcomes (cardiovascular death, non-fatal MI, non-fatal stroke, or late revascularization)

Among patients with obstructive CAD, the cumulative incidence of MACEs was highest in the early revascularization group, followed by no treatment and aspirin alone groups [35, 32, and 30 per 1000 patient-years, respectively] (see Table 2). Conversely, the lowest incidence was observed in the statins alone group, followed by the combination of statin and aspirin group [16 and 19 per 1000 patient-years, with *p* < 0.001 and *p* = 0.001, respectively].

For patients with non-obstructive CAD, the cumulative incidence of MACEs was highest for the early revascularization group [52 per 1000 patient-years], followed by the combination of statin and aspirin, and aspirin alone groups [14 and 13 per 1000 patient-years, respectively], and lowest in the statins alone and no treatment groups [7 and 10 per 1000 patient-years, respectively].

Interestingly, among patients with no coronary artery stenosis, the cumulative incidence of MACEs was highest in the aspirin alone, and the combination of statin and aspirin groups [18 and 14 per 1000 patient-years, respectively], and lowest in the statins alone and no treatment groups [3, and 5 per 1000 patient-years, respectively].

### Association between treatment modalities and MACE Occurrence

**Fig. 1:**
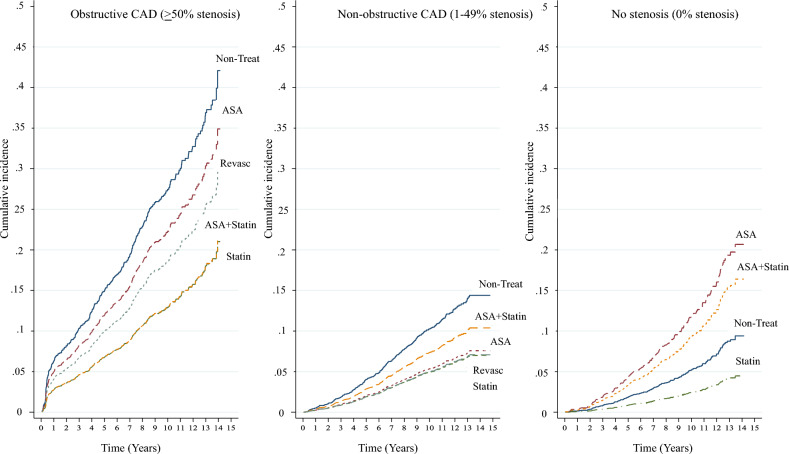
Cumulative incidence of MACEs by different treatment modalities grouped by degree of coronary stenosis on CCTA

To assess the relationships between different treatment modality and MACEs, a multivariate cause-specific hazard model was used which considered all significant clinical variables (from univariate analysis) stratified by the three CAD groups (Table 3). Cumulative incidence curves of MACEs were constructed with adjustment for confounding variables (Fig. 1). The curves reveal that the incidence of MACEs was lowest in patients treated with statins alone, irrespective of the presence/severity of coronary stenosis as determined by CCTA. These differences were evident in year 1 and persisted throughout follow-up, particularly in the obstructive CAD group.

**Table 3:** Hazard ratios of MACE occurrence by characteristics and treatment modalities grouped by degree of coronary stenosis: multivariate

	Obstructive CAD (≥ 50%), *n* = 1788			Non-obstructive CAD (1–49%), *n* = 4030			No stenosis (0%), *n* = 3520		
	HR	95% CI	*p* value	HR	95% CI	*p* value	HR	95%CI	*p* value
Treatments									
Early-revas	0.64	(0.43,0.97)	0.035	0.47	(0.07, 3.23)	0.444			
ASA + statin	0.43	(0.33,0.58)	< 0.001	0.71	(0.51, 0.98)	0.038	1.82	(1.20, 2.76)	0.005
Statin	0.43	(0.32, 0.58)	< 0.001	0.47	(0.34, 0.64)	< 0.001	0.46	(0.31, 0.69)	< 0.001
ASA	0.79	(0.41, 1.52)	0.643	0.51	(0.18, 1.43)	0.200	2.35	(1.04, 5.30)	0.040
No-medication	1			1			1		
CACS									
≥ 400	3.32	(1.86, 5.92)	< 0.001	3.31	(2.03, 5.38)	< 0.001			
100–399	2.83	(1.61, 4.97)	< 0.001	1.53	(1.04, 2.23)	0.029	0.74	(0.18, 3.02)	0.671
1–99	1.81	(1.01, 3.22)	0.045	1.13	(0.84, 1.52)	0.432	1.40	(1.00, 1.94)	0.047
0	1			1			1		
HT									
Yes	3.31	(2.12, 5.16)	< 0.001	3.62	(2.32, 5.64)	< 0.001	2.14	(1.47, 3.12)	< 0.001
No	1			1			1		
DM									
Yes	1.46	(1.17, 1.81)	0.001	1.60	(1.23, 2.08)	< 0.001			
No	1			1					
Number of stenotic vessels									
3 vessels/LM	1.48	(1.08, 2.01)	0.014						
2 vessels	1.20	(0.92, 1.56)	0.181						
1 vessel	1								
CKD									
eGFR < 60				1.57	(1.11, 2.21)	0.011	2.01	(1.24, 3.27)	0.005
eGFR ≥ 60				1			1		
CAVI									
≥ 9				1.65	(1.28, 2.12)	< 0.001	1.39	(1.04, 1.86)	0.027
< 9				1			1		

Abbreviations as in Table 1

HR and MACEs: see Table 2

After adjusting for confounding variables, patients receiving statins alone in the obstructive CAD, non-obstructive CAD and no coronary artery stenosis groups were less likely to develop MACEs compared to those who did not receive any treatment. The hazard ratios (95% CI) for the three groups were 0.43 (0.32, 0.58), 0.47 (0.34, 0.64), and 0.46 (0.31, 0.69), respectively (Table 3).

In the obstructive and non-obstructive CAD groups, patients treated with a combination of statin and aspirin had HRs (95% CI) of 0.43 (0.33, 0.58) and 0.71 (0.51, 0.98), respectively, showing a significantly lower likelihood of developing MACEs compared to the no treatment group. In patients with obstructive CAD who underwent revascularization, the HR was 0.64 (0.43, 0.97), indicating a reduced risk of events compared to the no treatment group. Conversely, in patients with no coronary artery stenosis, treatment with aspirin alone, and a combination of statin and aspirin, were associated with a higher likelihood of developing MACEs compared to no treatment [HRs (95% CI) of 2.35 (1.04, 5.30) and 1.82 (1.20, 2.76), respectively] (Table 3).

In addition to treatment modality, several other clinical variables were significantly associated with the incidence of MACEs. Importantly, in patients with non-obstructive CAD or no coronary artery stenosis there exists a possibility of plaque progression in a coronary artery which can subsequently result in a CV event. The patients in our cohort who developed MACEs tended to have a higher prevalence of ASCVD risk factors, such as hypertension, DM, and CKD, as well as coronary artery calcium and arterial stiffness [as detected by a high CAVI (≥ 9)].

## Discussion

This cohort study investigated the associations between different treatment modalities guided by CCTA findings and the occurrence of long-term MACEs in patients at risk for CV disease. Our findings support those of the PROMISE trial [23] SCOT-HEART trial [6] and Coronary CT Angiography Evaluation for Clinical Outcomes: An International Multicenter (CONFIRM) registry [24], which all demonstrated the potential benefit of CCTA in guiding appropriate treatment to prevent CV events. CCTA provides detailed images of the coronary arteries, allowing for detection and assessment of extent and severity of CAD. Analysis of these images allows the personalizing of therapy and identification of high-risk patients who may benefit from special interventions such as revascularization.

To our knowledge, randomized controlled studies comparing different CCTA-guided treatment modalities to prevent future MACEs in both asymptomatic individuals with CV risk factors and symptomatic patients with stable CAD are lacking. This is especially true when comparing different modalities of treatment such as aspirin, statin, or early revascularization. Our findings indicate that different treatment modalities guided by CCTA findings appears to be associated with varying preventive effects on MACEs. However, it is important to note that during the study enrollment period (2005–2013), predating the release of the 2016 Coronary Artery Disease-Reporting and Data System (CAD-RADS) Expert Consensus Document [25], a standardized approach for utilizing CCTA to allocate treatment was absent. Consequently, there was no agreed upon decision tree to guide specific treatment based on coronary anatomy results. Patients were managed according to their physician’s judgment, utilizing CCTA findings alongside other findings such as coronary artery calcium score, cardio-ankle vascular index for arterial stiffness (as per our protocol), and other risk factors. Our study was observational, noting the actual treatments utilized after CCTA findings were available. These preliminary results from real-world practice elucidated the CCTA’s effect on treatment selection, particularly in the Asian context, and contributed some insights to this unresolved matter.

The presence and extent of coronary artery plaque, as assessed by CCTA, were found to be strong predictors of future CV events. Subclinical atherosclerotic plaques identified by CCTA, whether obstructive or non-obstructive CAD, place patients in priority groups for preventive therapy. During a follow-up period averaging 10 years, patients with obstructive CAD had the highest frequency of MACEs (about 20%), compared with those with non-obstructive CAD (10%) and no coronary artery stenosis (5%). This is consistent with previous observational studies that reported three to fourfold and twofold increases in risk of MACE for obstructive CAD and non-obstructive CAD relative to no coronary artery stenosis based on CCTA [26–28]. Most of the CV events in the patients of the three of our groups were non-fatal strokes and MIs, as well as revascularizations in the obstructive CAD group. However, the rates of CV death were quite low (0.3–1.2%) across the three groups, likely explained by widespread use of statins (about two-thirds) in our cohort.

Statins have been used as a cardiovascular protective therapy and are recommended for primary prevention. In this cohort statins alone were associated with lower incidences of long-term MACEs in all three patient groups, irrespective of the severity of coronary stenosis determined by CCTA. It is possible that patients on statin treatment received better care or were more health-conscious, and thereby contributed to the reduction in long-term MACEs. However, our retrospective review revealed an under-prescription of statins for patients with obstructive and non-obstructive CAD, while there was an overutilization in patients without coronary stenosis. Importantly, a quarter of patients with obstructive CAD and a third with non-obstructive CAD did not receive any medical therapy, despite positive coronary artery findings on CCTA. Possible explanations for these findings include treatment decisions made by a diverse group of physicians (and perhaps not cardiologists), a lack of clear consensus on appropriate treatment, and the patient’s socioeconomic and personal preferences. Recent studies have demonstrated the effectiveness of statins in reducing CV morbidity and mortality, slowing the progression of atherosclerosis and stabilizing plaques [29–32]. A protective role of statins on clinical outcomes has also been demonstrated in patients with non-obstructive CAD or even those with no coronary artery stenosis [33, 34]. Other reports differed in that statin treatment was shown to improve survival only in patients with obstructive or non-obstructive CAD, but not in those with normal angiograms [35–37]. Current international guidelines [38] recommend statin treatment for all patients with intermediate ASCVD risk (≥ 7.5 to < 20% 10-year risk), and for patients with chronic coronary syndrome, including those with microvascular angina or cardiac syndrome X [9].

While the benefit of statin therapy is well established for primary prevention, the role of aspirin remains controversial due to the lack or only marginal benefit observed, while it carries a significant bleeding risk [39–43]. The recent 2019 American College of Cardiology/American Heart Association (ACC/AHA) primary prevention guidelines suggest that aspirin may be considered in selected adults aged 40–70 years with higher CV risk but not increased bleeding risk (class IIb) [38]. In our study, aspirin appeared to have a benefit in patients with both obstructive and non-obstructive CAD (as detected by CCTA) with risk reductions of 29% and 49%, respectively, relative to no-medication, but not in patients without stenotic lesions. In addition, the effect of ASA was neither superior to statins alone nor different between patients with obstructive and non-obstructive CAD, as the 95% CIs of ASA effect of these two groups overlapped [(0.41, 1.52) and (0.18, 1.43), respectively].

Importantly, aspirin was associated with increased MACE outcomes in the no stenosis group (or normal CCTA), whether given alone or in combination with statin. The reason of this is not clear, but there are several possible explanations. First, this study was observational and could not establish cause-and-effect relationships between medications and outcomes. Second, aspirin may cause bleeding events which can overall be more harmful than beneficial in patients with infrequent CV events. Third, aspirin may not be effective at preventing ischemic events in patients with normal CCTA. Last, the number of patients treated with statin combined with aspirin or with aspirin alone in the no stenosis group was quite small, and so the power to see group differences was quite small. Thus, antiplatelet therapy is generally not recommended in such a low-risk group because the chance of bleeding may outweigh its benefit.

In patients with CAD suspected to be obstructive, CCTA can effectively detect high-risk CAD, especially triple-vessel or left main disease, and guide management decisions, including revascularization [9]. However, merely 7.3% of patients exhibited obstructive CAD according to CCTA findings and subsequently underwent revascularization. Several factors likely contributed to the underutilization of early revascularization in our cohort: (1) the majority of patients were asymptomatic or had only mild symptoms and had not received prior treatment, (2) the definition of obstructive CAD necessitated more than 50% diameter stenosis in at least one vessel, a less stringent threshold than the 70–99% stenosis generally used as an indication for revascularization. Interestingly, those patients who underwent early revascularization had a higher incidence of subsequent CV events than did those treated with statin alone. Such patients potentially had triple-vessel or left main disease, which correlated with an elevated occurrence of MACEs, or experienced in-stent restenosis or stent thrombosis following the revascularization, necessitating repeated revascularization procedures. Unfortunately, due to the need for re-adjudication of in-stent restenosis or stent thrombosis by interventional cardiologists, definitive information concerning these subsequent events could not be established.

Our findings support the recommendations of the Clinical Outcomes Utilizing Revascularization and Aggressive Drug Evaluation (COURAGE) [44] and International Study of Comparative Health Effectiveness with Medical and Invasive Approaches (ISCHEMIA) [45] clinical trials, that initial therapy be medical, and only if this fails would angiography with revascularization be considered. Our findings underscore the criticality of patient selection for early revascularization, particularly in those asymptomatic or whose symptoms are mild and stable. It’s worth noting that only 5.1% of patients with obstructive CAD underwent cardiac stress testing to evaluate the hemodynamic significance of coronary lesions. In truth, a more comprehensive hemodynamic assessment should be routinely administered to this cohort of patients. Nowadays, as per the CAD-RADS™ 2.0-2022 consensus [46] and 2023 guidelines for the management of patients with chronic coronary syndrome [47], patients with stable chest pain should be considered for functional assessment or invasive coronary angiography if maximal coronary stenosis is above 50%. Recently, CT-derived functional flow reserve (CT-FFR) measurement has further refined the role of CCTA in decision-making regarding revascularization, optimizing more benefit and avoiding unnecessary treatments.

Given the retrospective design of this study, it is important to address the concern that during long duration of follow-up there may have been plaque progression leading to symptomatic CAD or stroke in patients grouped as non-obstructive or no coronary artery stenosis based on CCTA findings [48]. In this study the percentages of individuals having non-fatal MIs and strokes within the non-obstructive CAD and no coronary artery stenosis groups were 0.7 and 2.0%, and 4.5 and 6.9%, respectively. Those who developed a MACE tended to have more ASCVD risk factors (such as hypertension, DM, and CKD, along with elevated CACSs and CAVIs). In this study, we found that a high CAVI was an independent risk predictor of a MACE, especially in patients with non-obstructive CAD or no coronary artery stenosis. To prevent the occurrence of MACEs, our evidence suggested that all individuals with mild symptomatic CAD, or positive risk factors even when asymptomatic, should be treated at least with statins alone, regardless of the CCTA findings. This strategy may be effective and adequate for primary prevention of CV events across all vascular territories without incurring risk of bleeding [43, 49].

In summary, this retrospective cohort study demonstrated that CCTA offers useful guidance for the treatment of patients with stable CAD and shows potential for prevention of CV events. However, a definitive assessment of the efficacy of adding CCTA findings into CAD management decisions is not possible from this retrospective analysis. Nevertheless, the analysis of this large cohort allows for preliminary conclusions. The full validation of a given strategy utilizing CCTA will likely require a prospective longitudinal study, utilizing a randomized clinical trial design.

### Study limitations

Despite this study having a large cohort and long follow-up, certain limitations should be acknowledged. First, the analysis did not consider individual patient symptoms at baseline due to incomplete records and inability to adjudicate. However, it is worth noting that most of our patients had only mild symptoms, unrelated to MI or acute coronary syndrome. Second, there was no available data concerning CAD-RADS score. Our study was conducted from 2005 to 2013, and the analyses of CCTA were completed before the first CAD-RADS 2016 consensus [46] was introduced. As a result, no grading scale for assessing plaque burden (segment involvement score) and detecting ischemia was utilized, apart from evaluating the extent of luminal diameter stenosis. Consequently, our findings could not be directly compared to other studies that utilized this score system. Third, all study endpoints identified during follow-up were obtained from medical records, reimbursement systems, and government IT databases, rather than prospectively collected from patients. As a result, there is a possibility that certain CV events were under-reported. Nevertheless, follow-up of the major outcome, death, was complete. Fourth, the risk at baseline of subsequent MACEs for patients receiving different treatment modalities is potentially confounded by factors that can influence clinical outcomes. Although we controlled for well-known confounding variables through statistical analyses, our findings may still have been impacted by other (known and unknown) confounders given our retrospective cohort design. These limitations would be best addressed through use of randomized clinical trials. Fifth, given the extended duration of follow-up in our study, adjustments in both dosage and type of statins occurred over time. This dynamic aspect led to the unavailability of comprehensive data regarding the specific effective of different types and varying dosages of statins in influencing future MACEs. Last, it is important to note that our cohort was not ethnically diverse, as all participants were Asian. Therefore, disparities observed between studies could, in part, be attributed to ethnic differences. Yet this potential limitation is also one of the strengths of the study, as the type of data generated and analyzed here is quite lacking for Asian populations.

## Conclusion

The severity of coronary stenosis, as assessed by CCTA, offers useful guidance for the treatment of patients with stable CAD and shows potential for prevention of CV events. Statin therapy alone was associated with a lowering of the incidence of MACEs, irrespective of the severity of coronary stenosis. In patients with obstructive CAD, the addition of aspirin or possible revascularization appeared to result in fewer CV events. However, a definitive assessment of the efficacy of adding CCTA findings into CAD management decisions is not possible from this retrospective analysis. The full validation of a given strategy utilizing CCTA will likely require a prospective longitudinal study, utilizing a randomized clinical trial design.

### Supplementary Information

Below is the link to the electronic supplementary material.

Supplementary file1 (DOCX 20 KB)

## Data Availability

The data support the findings of this study are available from the corresponding author upon reasonable request.
