# Supplementary material for: Impact of coronary CT angiography in selection of treatment modalities and subsequent cardiovascular events in Thai patients with stable CAD
Source: Clin Res Cardiol. 2023 Oct 4;113(3):433–45. doi: 10.1007/s00392-023-02313-1 (PMC10881602; doi:10.1007/s00392-023-02313-1)
Supplement: Supplementary file 1 — Supplementary file1 (DOCX 20 KB) [file 392_2023_2313_MOESM1_ESM.docx]

**Supplemental Table 1.** Incidence of long-term MACEs grouped by degree of stenosis on CCTA.

| Outcomes | Incidence (%)  (n=9338) | Obstructive CAD  (>50%)  N=1788 | Non-obstructive CAD  (1%-49%)  N=4030 | No stenosis (0%)  N=3520 | P value |
| --- | --- | --- | --- | --- | --- |
| MACEs | 10.3 | 19.8 | 10.3 | 5.5 | < 0.001 |
| CV death | 0.7 | 1.2 | 0.8 | 0.3 | < 0.001 |
| Non-fatal MI | 2.3 | 6.4 | 2.0 | 0.7 | < 0.001 |
| Non-fatal stroke | 6.2 | 8.1 | 6.9 | 4.5 | < 0.001 |
| Late revascularization | 3.4 | 10.5 | 2.7 | 0.6 | < 0.001 |
| GI bleeding +Intracranial bleeding | 2.5 | 4.0 | 2.7 | 1.6 | < 0.001 |

CV: cardiovascular, GI: gastrointestinal, MACEs: major adverse cardiovascular events (CV death or non-fatal MI or non-fatal stroke or late revascularization), MI: myocardial infarction
